# A novel paradigm to study interpersonal threat-related learning and extinction in children using virtual reality

**DOI:** 10.1038/s41598-017-17131-5

**Published:** 2017-12-04

**Authors:** Hilary A. Marusak, Craig A. Peters, Aneesh Hehr, Farrah Elrahal, Christine A. Rabinak

**Affiliations:** 1 Department of Pharmacy Practice, Wayne State University College of Pharmacy and Health Sciences, Detroit, MI USA; 2 Department of Pharmaceutical Sciences, Wayne State University College of Pharmacy and Health Sciences, Detroit, MI USA; 3 Department of Psychiatry and Behavioral Neurosciences, Wayne State University School of Medicine, Detroit, MI USA

## Abstract

Disruptions in fear-extinction learning are centrally implicated in a range of stress-related disorders, including anxiety and posttraumatic stress disorder. Given that these disorders frequently begin in childhood/adolescence, an understanding of fear-extinction learning in children is essential for (1) detecting the source of developmental susceptibility, (2) identifying mechanisms leading to pathology, and (3) informing the development and/or more judicious application of treatments for youth. Here, we offer and validate a novel virtual reality paradigm to study threat-related learning and extinction in children that models real-world cues, environments, and fear-inducing events that children are likely to experience, and are linked to the development of fear- and stress-related pathologies. We found that our paradigm is well tolerated in children as young as 6 years, that children show intact fear and extinction learning, and show evidence of divergence in subjective, physiological, and behavioral measures of conditioned fear. The paradigm is available for use in 3-D and in 2-D (e.g., for the MRI scanner) upon request at www.tnp2lab.org.

## Introduction

Disruptions in fear and extinction learning are considered central to the development of posttraumatic stress disorder (PTSD) and other fear- and stress-related psychopathologies^1^. Given that these disorders frequently begin in childhood and adolescence^2^, an understanding of fear and extinction learning and the underlying neurobehavioral mechanisms in children/adolescents (youth) is essential for (1) detecting the source of developmental susceptibility, (2) identifying mechanisms leading to pathology, and (3) informing the development and/or more judicious application of treatments for youth. Further, early alterations in fear-extinction processes might help to explain (4) why adults with histories of exposure to fear-inducing events during childhood (e.g., violence, abuse) are more susceptible to stress-related pathologies.

Fear conditioning and extinction processes have been examined in the context of Pavlovian fear conditioning paradigms, which traditionally involve pairing of a previously innocuous cue (conditioned stimulus, CS) with an aversive outcome (unconditioned stimulus, US). After repeated pairings with the US, presentation of the CS begins to elicit a conditioned fear response, which is typically measured by physiological indices such as skin conductance response (SCR) and/or subjective ratings of fear or US expectancy. After repeated presentations of the CS in the absence of the US, however, a new memory is formed that competes with the fear memory trace - so-called “extinction learning”^3^. Extinction represents the theoretical basis of exposure-based treatments for fear- and stress-related pathologies (e.g., cognitive-behavioral therapy [CBT]) that were developed for adults^4^, and are commonly applied to children and adolescents.

Given that fear and extinction learning are considered central to the pathophysiology and treatment of fear-based disorders, it is essential to have age-appropriate paradigms to study fear-extinction in children and adolescents. It is also critical that the paradigm model real-world cues, environments, and fear-inducing events that children are likely to experience, and are linked to the development of fear- and stress-related pathologies. Interpersonal threat exposures (e.g., community or domestic violence, abuse, assault) are extremely common during childhood and adolescence; national survey data indicate that over 70% of youth will be victims of violence, crime, and/or abuse before their 18^th^ birthday^5^. Further, exposure to interpersonal threat during childhood has been strongly linked to the development of virtually all commonly-occurring fear-related disorders (i.e., PTSD, anxiety^2,6^). With these data in mind, we developed a novel fear-extinction paradigm for children that involves cues that are relevant for interpersonal threat, and incorporates relevant real-world cues and environments that children commonly experience. To do this, we adapted a standard Pavlovian cued fear conditioning-extinction paradigm with contextual modulations^7^ by implementing virtual reality (VR). Virtual people (‘avatars’) were chosen as CSs because they are relevant for modeling the interpersonal nature of common fear-inducing events, and the incorporation of immersive virtual environments may allow for robust manipulation of environmental contexts. An aversive white noise burst (500 ms, 95 dB, at 75% reinforcement) served as the US. Selection of the US was based on a decision to balance tolerability of the US for young participants with the ability to elicit robust fear responses. Prior pediatric studies have investigated the use of different stimuli as the US, for e.g., an aversive auditory scream, and have observed relatively high discontinuation rates (up to 49% among anxious youth^8,9^).

**Figure 1:**
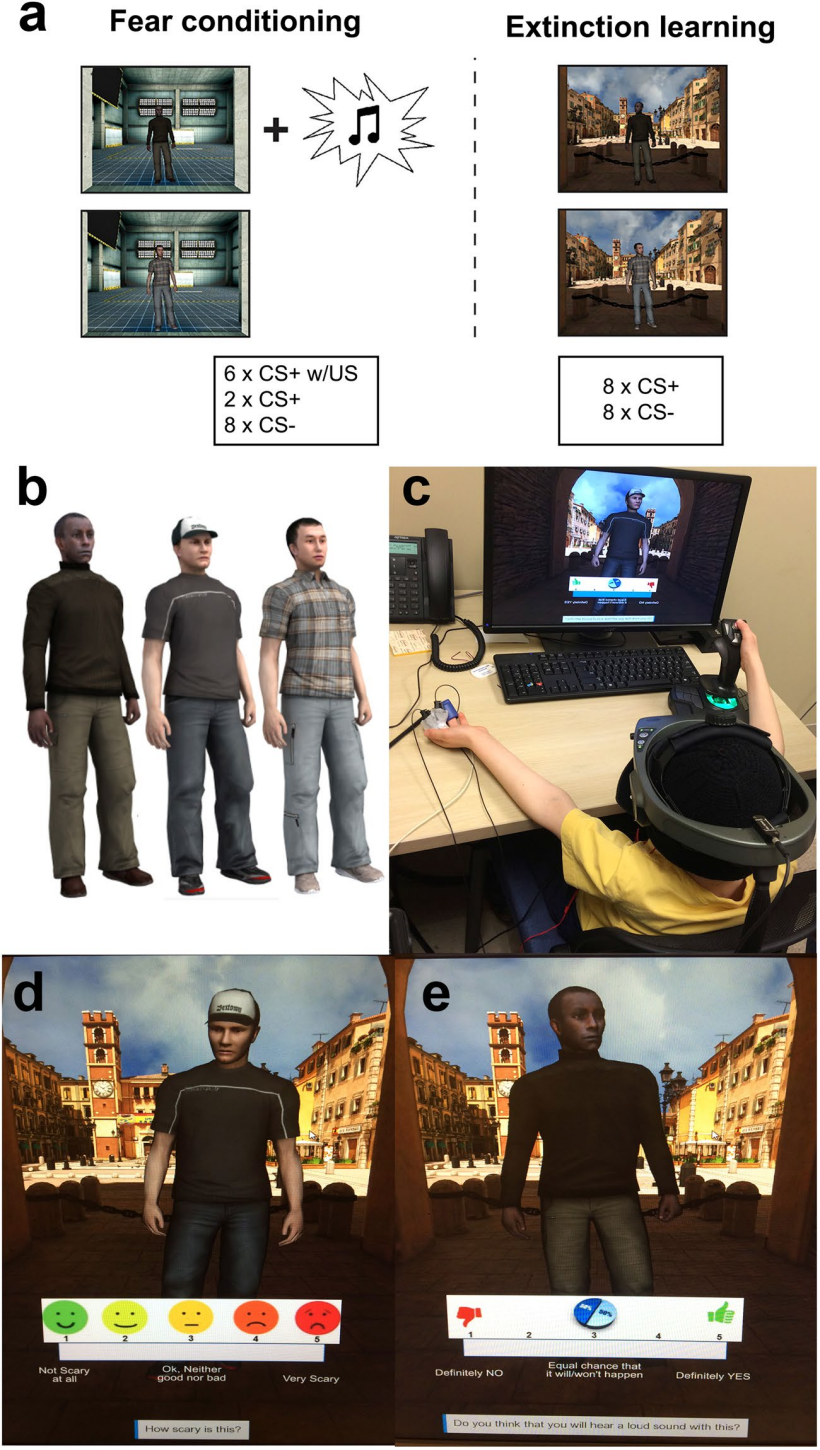
Virtual reality interpersonal threat-related learning and extinction paradigm. (**a**) Fear conditioning occurred in a “danger” context (CXT+) and extinction learning subsequently occurred in another, “safety” context (CXT−), separated by 10 min. A white noise burst served as the unconditioned stimulus (US). Trial numbers are provided below the stimuli - derived from prior pilot versions of the task in children. (**b**) Virtual adult males served as conditioned stimuli (CS), because they are relevant for modeling the interpersonal nature of common threat exposures (e.g., violence, abuse) that children frequently experience. CSs varied in race/ethnicity, to match the varied demographics of the study sample. Designation of virtual contexts and people to the danger/safety context and CS type, respectively, were counterbalanced across participants. (**c**) Child completing the experiment in virtual reality. Children actively navigate within the virtual environment and submit fear and US expectancy ratings using a joystick in their dominant hand. Skin conductance response (SCR) data are recorded in the non-dominant hand. (**d**) Example fear rating screen: “How scary is this?”. (**e**) Example US expectancy rating screen: “Do you think you will hear a loud sound with this?”. Stimuli and simulations were created using the WorldViz VR Toolkit (Developer Edition; www.worldviz.com) and included here, with permission.

In this initial study, we aimed to validate our new paradigm (see Fig. 1) in a sample of N = 43 children, ages 6–11 years. The overall sample is considered at high sociodemographic risk for stress-related pathologies (i.e., lower income, urban dwelling^10^), however a subset of the 43 children were additionally recruited for exposure to violence, abuse, or other fear-inducing events (e.g., intensive medical treatments). Thus, this initial validation study includes our population of interest (i.e., children with histories of exposure to fear-inducing events). We tested the tolerability of our novel paradigm in children as young as age 6, and evaluated whether children could acquire fear associations (i.e., show fear conditioning) and subsequently extinguish conditioned fear responses during an extinction learning session. To compare our results with previous studies, we focused on fear conditioning and extinction learning in the present study. Based on existing studies in children and in rodent models^11,12^, we predicted that both fear conditioning and extinction would be intact by age 6, and that relative to older children, younger children would show poorer ability to discriminate between cues and/or would be more likely to generalize fear across cues^13,14^. SCRs and fear and US expectancy ratings served as physiological and subjective indicators of fear, respectively, as commonly used in fear-extinction studies. However, in our paradigm, children are also given the opportunity to interact with their environment in the context of fear conditioning-extinction paradigm – something that has not been measured in previous pediatric fear-extinction studies. Thus, we examined multiple levels of conditioned fear responses via self-report, physiological, and behavioral action tendencies (approach/avoidance). Given that fear is made up of different dimensions (e.g., subjective, physiological, behavioral) that likely map on to different symptoms of fear-related pathologies (hyper-arousal, distress, avoidance), we expected to see both areas of convergence and divergence among these measures – similar to previous Pavlovian fear-extinction studies in adults e.g.,^15^. We also explored patterns of age-related change in these measures.

## Results

### Discontinuation rates

While one of the N = 43 participants (prior exposure to threat) chose to discontinue during fear conditioning, she did elect and was able to complete the paradigm on the computer screen rather than with the VR headset. Thus, data from all 43 participants were useable.

### Subjective measures of conditioned fear - Fear and US expectancy ratings

**Figure 2:**
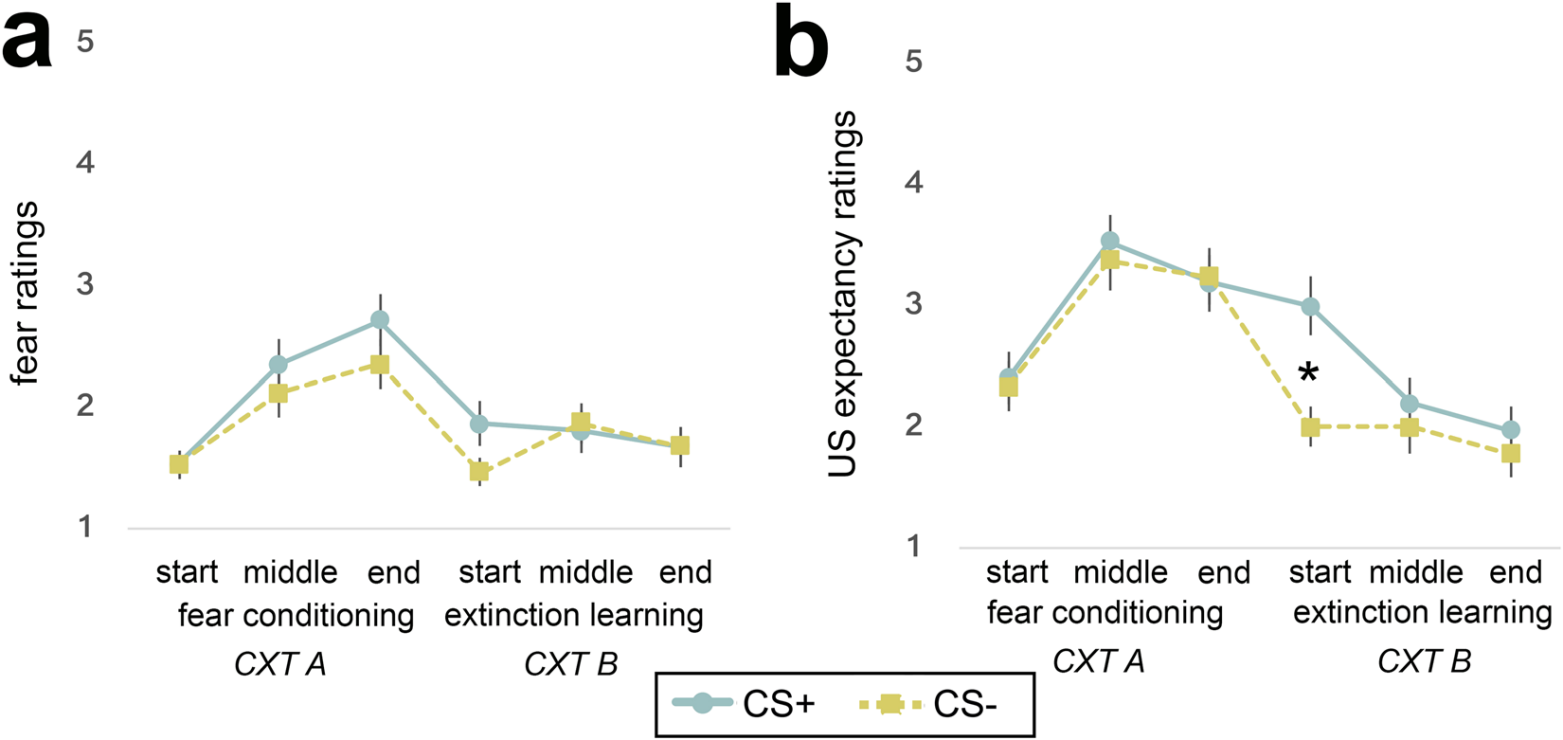
Fear and US expectancy ratings during fear conditioning and extinction learning in children. Significant increase in fear (**a**) and US expectancy ratings (**b**) for the CS+ but not the CS− over the course of fear conditioning, suggesting intact conditioning. At the start of extinction, US expectancy ratings were significantly higher for the CS+ relative to the CS−, further suggesting differential conditioning. During extinction learning, there was a decline in fear and US expectancy ratings for the CS+ and the CS−, suggesting intact extinction. Fear (“How scary is this?”, 1 = not scary, 5 = very scary), and US expectancy (“Do you think that you will hear a loud sound with this?”, 1 = definitely not, 5 = definitely yes) ratings are submitted at the beginning (‘start’), after the first half (‘middle’), and at the end (‘end’) of each session. CS+ and CS− refer to the avatar (virtual person) that is paired and unpaired with the US (white noise burst), respectively. Error bars represent standard error. Abbreviations: CS, conditioned stimulus; US, unconditioned stimulus; CXT, context.

Fear and US expectancy ratings are shown in Fig. 2. During fear conditioning, there was a significant main effect of time (start [before first trial], end [after last trial]; *F*[1,14] = 8.3, *p* = 0.012, η_p_^2^ = 0.37) and a significant time x CS-type (CS+, CS−) interaction (*F*[1,14] = 10.33, *p* = 0.006, η_p_^2^ = 0.425) for fear ratings. Paired-sample *t*-tests showed that the interaction was driven by a significant increase in fear ratings to the CS+ (*p* = 0.002, Cohen’s *d* = 1.2) - but not to the CS− (*p* = 0.21) - from the beginning to the end of fear conditioning. The significant increase in fear ratings for the CS+ but not CS− over the course of conditioning supports intact conditioning (Fig. 2a). The main effect of time (*p* = 0.41) and the time x CS-type (CS+, CS−) interaction (*p* = 0.096) did not reach significance for US expectancy ratings during conditioning. Although the difference in fear and US expectancy ratings between CS+ and CS− did not reach significance at the end of the conditioning session (*p* = 0.087 and *p* = 0.84, respectively) or at any point during conditioning, there was a significant difference at the start of extinction learning such that children reported greater US expectancy for the CS+ relative to the CS− (*p* = 0.013, Cohen’s *d* = 0.76; see Fig. 2b), further supporting differential conditioning. Variation in observed patterns for fear and US expectancy ratings is not surprising, given that fear and expectancy ratings are thought to capture different dimensions of learning^16^. In particular, valence ratings are thought to reflect evaluative learning whereas expectancy ratings reflect signal learning^16^. During extinction, there was a significant main effect of time (start, end; *F*[1,14] = 6.2, *p* = 0.026, η_p_^2^ = 0.31) and a main effect of CS-type (CS+, CS−; *F*[1,14] = 7.1, *p* = 0.019, η_p_^2^ = 0.37) for US expectancy ratings, indicating a significant decline in expectancy ratings over the course of extinction and higher overall expectancy ratings to the CS+ relative to the CS− (Fig. 2b). For fear ratings, there were no significant main effects or interactions during extinction (*p*’s > 0.09). However, there were significant declines in fear ratings from the end of conditioning to the end of extinction (*p’s* < 0.01).

### Physiological measures of conditioned fear - Skin conductance responses (SCR)

**Figure 3:**
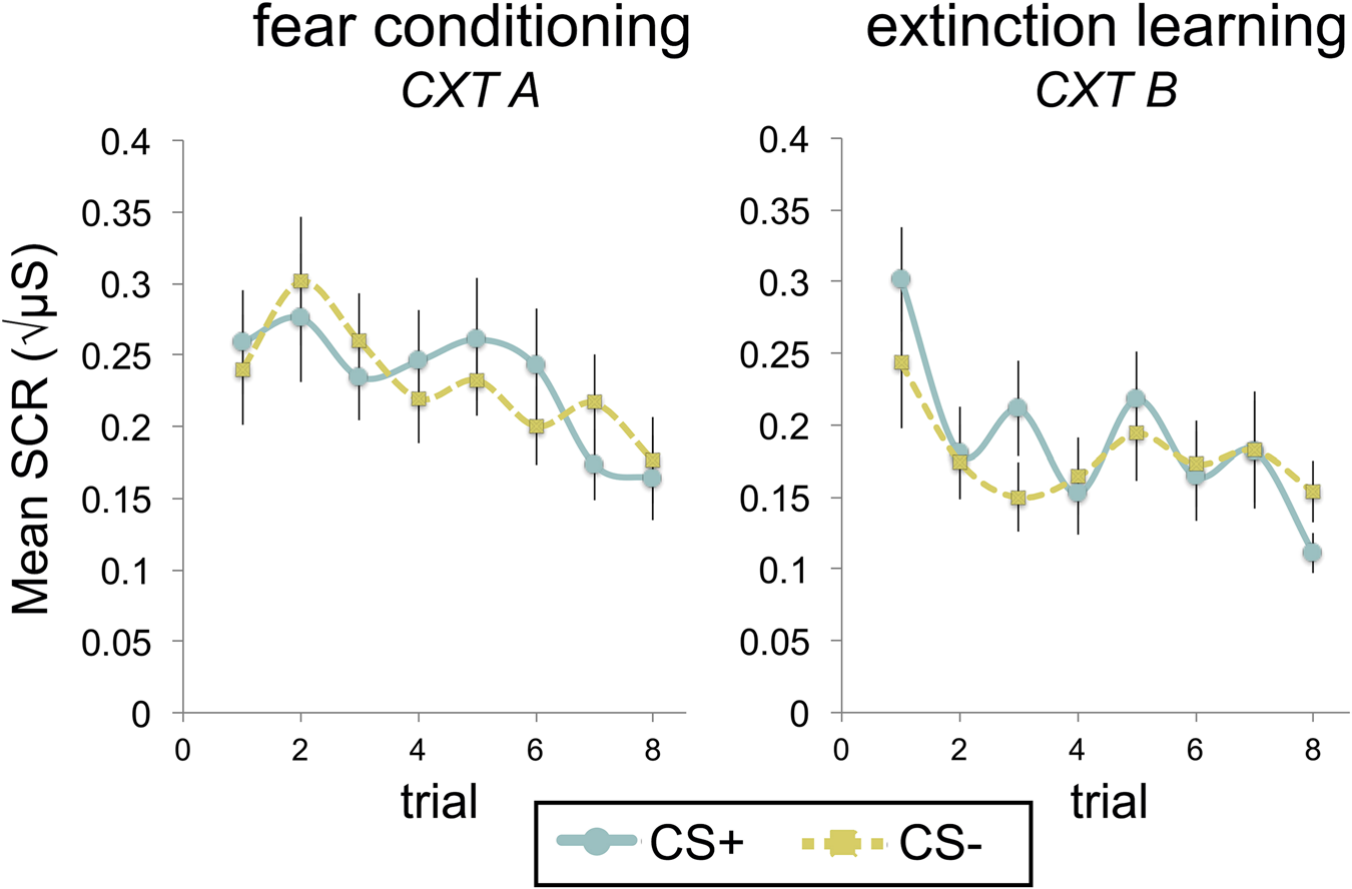
Skin conductance response (SCR) during fear conditioning and extinction learning in children. CS+ and CS− refer to the avatar (virtual person) that is paired and unpaired with the US (white noise burst), respectively. SCRs to both the CS+ and CS− declined during fear conditioning, consistent with previous fear conditioning studies in adults and in children^17–20^. Children show a significant decline in SCRs to the CS+ and the CS− during extinction, suggesting intact extinction learning. Error bars represent standard error. Abbreviations: CS, conditioned stimulus; US, unconditioned stimulus; CXT, context.

SCR data are shown in Fig. 3. During fear conditioning, there was a significant main effect of time (first trial, last trial; *F*[1,37] = 4.58 *p* = 0.039, η_p_^2^ = 0.11) such that SCRs were higher at the beginning than the end of fear conditioning. There was no significant main effect of CS-type (CS+, CS−) or significant time x CS-type interaction (*p*’s > 0.4). A decline in physiological indicators of conditioned fear over the course of fear conditioning is commonly reported in studies in both adults and children^17–20^, with some studies reporting a concomitant increase in subjective ratings (e.g.,^18^), as observed here. A decline in physiological responding over time is thought to reflect habituation^21^. The common observation of divergence in fear indicators has prompted calls for including multiple measures of conditioned fear in studies of fear conditioning, as these indicators may represent separate dimensions of fear learning and divergence between measures and individual differences therein may be linked to pathology^22,23^. As expected, average SCRs to the US (i.e., the unconditioned response, 0.45 ± 0.36 √µS) were significantly higher than SCRs to the CS+ (i.e., the conditioned response; *p* < 0.001, Cohen’s *d* = 0.99). During extinction learning, there was a significant main effect of time (first trial, last trial; *F*[1,36] = 19.94, *p* < 0.001, η_p_^2^ = 0.36) and a significant time x CS-type (CS+, CS−) interaction (*F*[1,36] = 9.47, *p* = 0.004, η_p_^2^ = 0.21). Subsequent *t*-tests indicated that children successfully extinguished conditioned fear responses, as evidenced by lower SCRs to the last relative to the first CS+ trial of extinction (*p* < 0.001, Cohen’s *d* = 0.99). There was also a significant decline in SCRs to the CS− from the first to the last extinction trial (*p* = 0.01, Cohen’s *d* = 0.59). While there was no significant difference in SCRs to the CS+ vs. CS− during the first extinction trial (*p* = 0.21), SCRs were lower to the CS+ than the CS− during the last extinction trial (*p* = 0.037).

### Behavioral measures of conditioned fear - Approach/avoidance behavioral action tendencies

**Figure 4:**
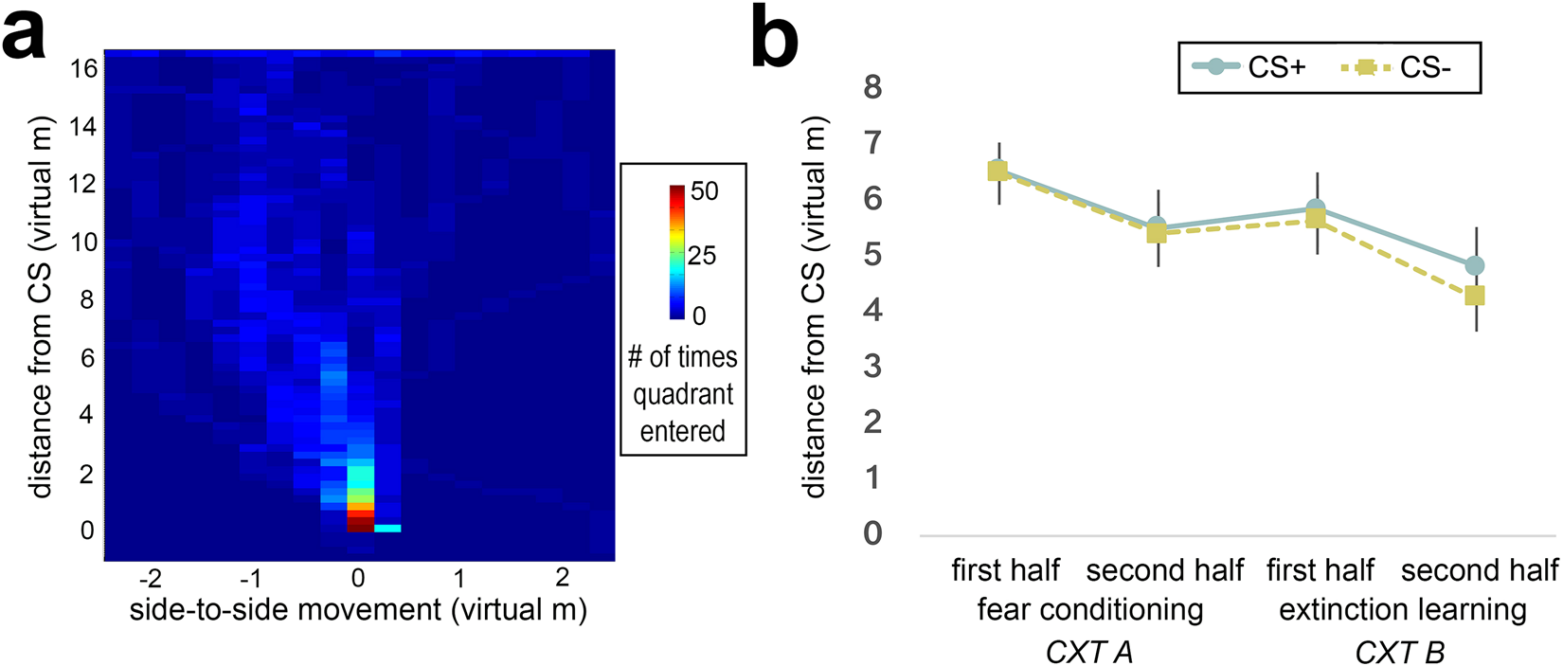
Approach/avoidant behavioral tendencies during fear conditioning and extinction learning in children. (**a**) Heatmap of movement in the virtual environment for an example participant during fear conditioning. Participant is placed at the starting point at the bottom of the image (x = 0, y = 0), and is instructed to move towards the conditioned stimulus (CS) at the top of the image. Warm and cool colors indicate number of times participant visited a quadrant. Distance from the CS (y-axis), as well as side-to-side distance traveled (x-axis), are quantified for each CS type (CS+, CS−) as a measure of approach/avoidance behavioral action tendencies and used in statistical analyses. Of note, for display purposes the frequency value of the origin was censored to consider periods when the participant was placed into the origin. (**b**) Participants approached both CS’s over the course of fear conditioning and extinction learning. CS+ and CS− refer to the avatar (virtual person) that is paired and unpaired with the US (white noise burst), respectively. Error bars represent standard error. Abbreviations: CS, conditioned stimulus; US, unconditioned stimulus.

An example heatmap image of a participant’s spatial movement following CS onset during the fear conditioning session is shown in Fig. 4a. An example video tracing a participant’s spatial movement during the fear conditioning session is provided as Supplemental Material (black lines trace movement in the CXT prior to CS onset; red lines trace movement in the CXT following CS onset). Quantification of spatial movement and distance data showed that children kept more distance from both CSs during the first half relative to the second half of conditioning, evidenced by a significant main effect of time (first half, second half; *F*[1,41] = 6.42, *p* = 0.015, η_p_^2^ = 0.135; Fig. 4b). There was no significant main effect of CS-type (CS+, CS−), or time x CS-type interaction for forward-to-backward motion. During extinction, the main effect of time was also significant, such that children kept more distance from both CSs during the first half relative to the second half of extinction (*F*[1,40] = 7.95, *p* = 0.007, η_p_^2^ = 0.166; Fig. 4b). Further, there was a positive correlation between distance from the CS+ during acquisition and extinction, *p*’s < 0.001, suggesting similar behavioral patterns between sessions. Taken together with the subjective and physiological data, these data indicate significant divergence among fear indicators.

### Age effects

Exploratory analyses tested for effects of age on subjective, physiological, and behavioral measures of conditioned fear responses during fear conditioning and extinction sessions. Increased age was associated with higher US expectancy ratings to the CS+ at the end of fear conditioning (*r*(42) = 0.34, *p* = 0.028), and with lower SCRs to the last CS− trial during fear conditioning, (*r*(40) = −0.38, *p* = 0.017), suggesting better differential conditioning in older youth. There were no other effects of age on subjective, physiological, or approach/avoidance behavioral measures to the CS+ or the CS− during either session.

### Associations between conditioned fear response measures

**Figure 5:**
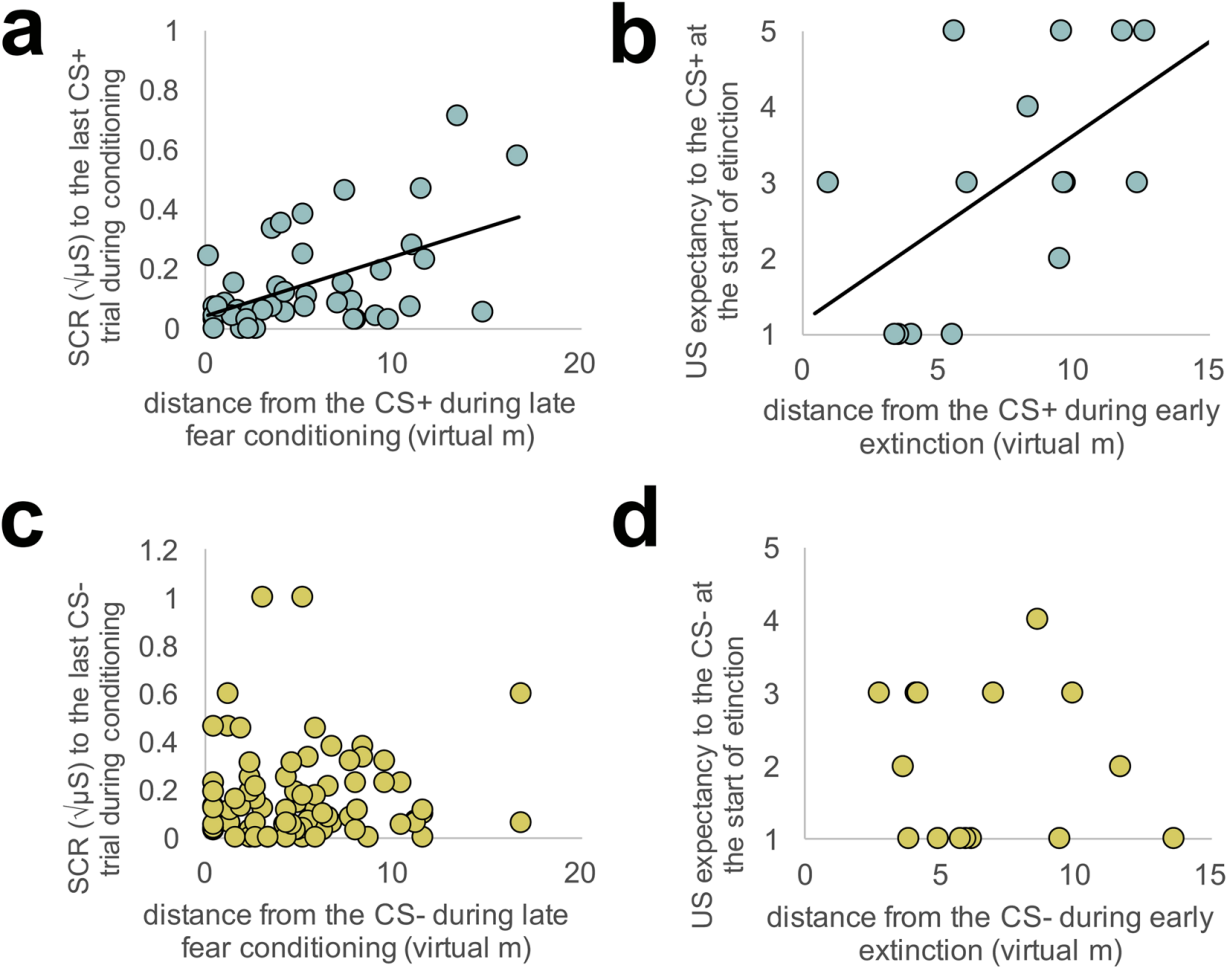
Inter-relations among subjective, physiological, and behavioral measures of conditioned fear. (**a**) Children who kept more distance from the CS+ during fear conditioning showed higher skin conductance response (SCR) to the last CS+ trial during conditioning, suggesting that avoidant behavior may predict higher physiological fear responding. (**b**) Children who reported higher US expectancy to the CS+ subsequently kept more distance from the CS+ during extinction. This suggest that CS−US contingency awareness may relate to avoidant behavior. (**c**,**d**) These associations were not significant for the CS−. CS+ and CS- refer to the avatar (virtual person) that was paired or unapired with the US (white noise burst), respectively, during fear conditioning. Abbreviations: CS, conditioned stimulus; US, unconditioned stimulus.

Exploratory analyses tested for correspondence among subjective, physiological, and behavioral markers of fear. Children who kept more distance from the CS+ during the first and second halves of fear conditioning demonstrated higher SCRs to the last CS+ conditioning trial (*r*[42] = 0.47, *p* = 0.002 and *r*[42] = 0.5, *p* = 0.001, respectively; Fig. 5a), suggesting an avoidant behavioral pattern may increase physiological markers of fear. These associations were not significant for the CS− (*p*’s > 0.15; see Fig. 5c). Children who reported greater US expectancy to the CS+ at the start of extinction learning subsequently kept more distance from the CS+ during the first half of extinction (*r*[15] = 0.57, *p* = 0.026; Fig. 5b), suggesting that subjective awareness may predict behavioral patterns. This association was not significant for the CS− (*p* = 0.57; Fig. 5d). Interestingly, there were no associations between subjective and physiological responses to the CS+ within either session (conditioning, extinction), suggesting significant divergence among typically measured fear indicators.

## Discussion

Here, we present a novel paradigm to study interpersonal threat-related fear conditioning and extinction learning in children using an immersive VR environment and dynamic stimuli. To our knowledge, this is the first application of a cued fear-extinction paradigm *within an environmental context* in a developmental sample. We developed this paradigm to include cues that are relevant for modeling the interpersonal nature of real-world threat exposures that children commonly experience (e.g., violence, abuse) and are strongly linked to the development of fear- and stress-related psychopathology. In our sample of 43 children, we found that our paradigm is well tolerated in children as young as age 6 (0% discontinuation). Further, many of the children included in the sample are considered at high risk for fear- and stress-related disorders (i.e., low-income families, exposure to threatening events), suggesting that our task is applicable to the pediatric population of interest. In line with the notion that VR is immersive and engaging, we find that our paradigm elicits robust fear conditioning, evidenced by increases in subjective measures of conditioned fear (i.e., fear and US expectancy ratings) over the course of conditioning. Children also show intact extinction learning, evidenced by declines in subjective and physiological (i.e., SCR) markers of conditioned fear. Importantly, we observed significant divergence in subjective, physiological, and behavioral (i.e., movement in the virtual environment) measures, in line with the notion that these indicators may represent separate dimensions of fear that may be sensitive to different aspects of pathology^22,23^. This highlights the potential utility of our paradigm in examining the development, maintenance, and treatment of fear-based disorders in children.

Several important findings emerged from our study. First, like previous pediatric fear-extinction studies, children showed intact fear conditioning and subsequent extinction learning, evidenced by increases in subjective ratings to the CS+ over the course of conditioning and subsequent declines during extinction learning. Interestingly, however we observed a divergence in physiological (i.e., SCR) and behavioral measures (i.e., movement) such that SCRs declined and children approached both CS’s over the course of fear conditioning. Previous studies in both adults and children have reported similar declines in physiological indicators of fear^17–20^. Subjective, physiological, and behavioral measures all declined during extinction learning, indicating intact extinction. Second, although there was evidence of differential conditioning in US expectancy ratings (i.e., higher US expectancy ratings for the CS+ relative to the CS−), there was no significant difference between CS+ and CS− in fear ratings, behavioral patterns, or SCRs. This suggests that fear learning may be generalized across cues (i.e., to the CS−) and/or children have difficulty in discriminating between threat and safety cues – at least in some response systems (e.g., physiology). Our data also suggest that differentiation ability increases with age. Overall, these patterns are consistent with prior pediatric fear conditioning studies^24,25^. Studies suggest that ability to discriminate between threat and safety cues increases with age in children^25^, and that children and adolescents may be more likely to generalize across cues than adults^13,14^. Generalization of fear may be relevant for the pathophysiology and treatment of anxiety disorders^26,27^.

Third, commonly-measured indices of conditioned fear (i.e., subjective ratings and SCRs) to the CS+ were not inter-related within fear conditioning or extinction sessions. Nonsignificant or weak inter-correlations among different conditioned fear indicators are frequently reported in fear conditioning studies in human. We are not the first to point out that divergence may reflect different components of fear that may map on to different symptom dimensions of fear-related pathologies (hyper-arousal, distress, avoidance)^22,28^. In addition to measuring subjective and physiological measures, our paradigm allows unique access to behavioral action tendencies (e.g., approach/avoidance), which have been largely ignored in existing fear conditioning studies^22^. This is a critical gap, given that behavioral action tendencies constitute a core dimension of fear, and that avoidance is a central feature of most fear- and stress-related disorders, which can be characterized as *behavioral* dysfunctions. Further, avoidant behavior may be a consequence of childhood threat exposures that increases risk for later psychopathology^29^ by maintaining or enhancing negative thoughts and emotions^30^. Avoidance also remains integral to the theoretical basis of behavioral interventions, such as cognitive behavioral therapy (CBT^31^). Thus, considering multiple dimensions of conditioned fear, as is accessible in our paradigm, may provide a more complete and nuanced understanding of fear learning and related pathologies.

Although subjective and physiological measures of fear to the CS+ were not inter-related during sessions, we did observe significant associations with behavioral responses. Namely, children who kept more distance from the CS+ during late fear conditioning subsequently demonstrated higher SCRs to the last CS+ conditioning trial. This suggests that a passive avoidant behavioral pattern (i.e., failure to approach the CS+) may lead to an increase in physiological fear responding. There was also an association between subjective ratings and behavior during extinction such that children who reported greater US expectancy to the CS+ at the start of extinction subsequently kept more distance from the CS+ during extinction. Taken together, these findings suggest that individual differences in behavioral action tendencies may not only represent another expression of conditioned fear, but may also serve to maintain or exacerbate fear. Thus, behavioral action tendencies may be relevant in understanding the pathogenesis of fear-related disorders, and may help to explain variation in treatment response.

One unique aspect of our paradigm is the ability to manipulate context. A key finding from the adult fear conditioning literature is that extinction is context-dependent^32^ – which is thought to explain some of the limitations of behavioral interventions that rely on principles of extinction learning (for e.g., renewal of fear when the CS is presented outside of the extinction context). Although these interventions are frequently applied downwards to pediatric populations, it is unknown if extinction is similarly context-dependent in children, and the role of context in modulating fear learning and expression. Although contextual modulation of learned fear and extinction was not the focus of the current study, the inclusion of a contextual component to the present paradigm allows for the examination of this mechanism in future studies. Indeed, greater understanding of contextual regulation of fear should inform the development and/or more judicious application of interventions for youth. In addition, the ability to include and manipulate contexts in our paradigm allows us to more closely model real-world fear-inducing events that children commonly experience (e.g., interpersonal violence) and are inexorably tied to environmental contexts. Contexts can play a critical modulatory role in fear and extinction learning by modifying the expression of fear, or signaling fear itself^32^.

Limitations of the study warrant mention. First, the present sample was limited to children, and therefore it is unclear if patterns observed between adolescents and adults using this paradigm would be similar to those observed during cued fear learning paradigms *without* a contextual environment (e.g.,^33^). Second, the VR headset used in the present study was developed for adults rather than children. The headset may have been uncomfortable for younger children, however all participants successfully completed the paradigm. Future studies should consider VR headsets specifically made for children. Third, there may be other physiological measures beyond SCR that may be more sensitive to conditioned fear learning. For example, heart rate variability offers a noninvasive indicator of autonomic nervous system activity, indexing both parasympathetic and sympathetic reactivity^34^.

In summary, the current study aimed to develop and validate a novel fear learning-extinction VR paradigm in children that includes cues that are relevant for modeling the interpersonal nature of common fear-inducing events that children frequently experience. Our paradigm also allows for manipulation of context and evaluation of robust conditioned fear at multiple levels (subjective, physiological, behavioral), and is well tolerated in children as young as age 6. The experimental task, which we have adapted for use in the magnetic resonance imaging (MRI) scan environment, is available upon request (www.tnp2lab.org).

## Methods

### Participants

This study reports on 43 racially and economically diverse children (ages 6–11), recruited for a larger study on early threat exposure (e.g., violence, abuse, intensive medical treatments) through community-based advertisements or referrals via healthcare providers in the Metro-Detroit area. Participant characteristics and demographics are presented in Table 1. Participants were screened for threat exposure prior to participation, using the parent report screener version of the Juvenile Victimization Questionnaire^35^. The focus of the present initial study was to provide a within-subject validation of our novel paradigm in the population of interest (i.e., children with prior exposure to fear-inducing events), and all within-subjects effects held when controlling for threat exposure. The Kaufman Brief Intelligence Test, Second Edition (KBIT-2^36^) was used to measure IQ and pubertal stage was determined according to self-reported Tanner stages questionnaire^37^. Of note, one child (no history of threat exposure) scored <70 on the IQ assessment. Results did not change when excluding this child from analyses. Study exclusion criteria consisted of brain injury that involved loss of consciousness or neurological condition (e.g., epilepsy) or non-native English speaker. Participants and their parents provided written informed assent/consent and all study procedures were approved by the Wayne State University Institutional Review Board (IRB). All study methods were performed in accordance with IRB guidelines and regulations.

**Table 1:** Sample characteristics and demographics.

Variable	n = 43
Age, m (SD)	8.9 (1.4)
Gender, n female (%)	22 (51%)
IQ, m (SD)	103 (15)
Pubertal development	
Pre/early (Tanner stages 1–2), n (%)	33 (77%)
Mid/late (Tanner stages 3–5), n (%)	10 (23%)
Annual income	
Less than $10,000, n (%)	5 (12%)
$10,000–20,000, n (%)	3 (7%)
$20,000–30,000, n (%)	8 (19%)
$30,000–40,000, n (%)	2 (4%)
$40,000–60,000, n (%)	4 (9%)
$60,000–100,000, n (%)	6 (14%)
$100,000–140,000, n (%)	8 (19%)
Over $140,000, n (%)	7 (16%)
Not reported, n (%)	
Race/Ethnicity	
Caucasian, n (%)	20 (47%)
African American, n (%)	16 (37%)
Hispanic, n (%)	2 (5%)
Asian American, n (%)	1 (2%)
Native American, n (%)	1 (2%)
Other	3 (7%)
Early Threat Exposure*	
Interpersonal threat (violence, abuse), n (%)	5 (11%)
Intensive Medical Treatment, n (%)	14 (33%)
*Acute lymphoblastic leukemia, n (%)*	*8 (57%)*
*Neuroblastoma, n (%)*	*4 (29%)*
*T-cell lymphoma*	*1 (7%)*
*Wilms tumor, n (%)*	*1 (7%)*
None, n *(%)*	24 (56%)

Abbreviations: n, number; m, mean; SD, Standard deviation. *Interpersonal threat exposure was assessed using the Juvenile Victimization Questionnaire.

### Procedures

#### Cued fear-extinction paradigm with contextual modulations

The task we developed was an adapted version of a well-validated Pavlovian cued fear-extinction paradigm developed by Milad and colleagues^7^, which manipulates context using an AB design. In this design, the context in which fear is first acquired (‘danger’ context; CXT+) is different from the context in which fear is subsequently extinguished (‘safety’ context; CXT−). Two different 3D immersive VR environments that were matched on size and layout, but comprised of different colors, textures, sounds (ambient noise), and background scenes, constituted the contexts (Fig. 1a). The conditioned stimuli (CSs) were three virtual adult males. Human avatars were chosen as cues because they are relevant for modelling the interpersonal nature of early threat exposures that children commonly experience (i.e., violence, abuse). The CSs varied in hair color/style, clothing, and race (African American, Asian, Caucasian) to match the demographics of the study sample (Fig. 1b). Of note, the CSs moved naturally in a dynamic fashion throughout the experimental paradigm (e.g., walked into the field of view at the end of the virtual hallway space and turned to face the participant; looked in different directions; swayed). The unconditioned stimulus (US) was an aversive white noise burst (500 ms, 95 dB, at 75% reinforcement), a stimulus that is commonly used in pediatric studies (for a review, see^38^). Of note, the VR fear-extinction paradigm was conducted between the hours of 12:00 PM and 4:00 PM to control for time-of-day effects. Please see the Supplementary Information for details about the paradigm and the advanced VR system.

#### Measures of Conditioned Fear

Subjective, physiological, and behavioral measures of conditioned fear were recorded. Subjective ratings were submitted at the beginning (‘start’), after the first half (‘early’), and after the second half (‘late) of each session (fear conditioning and extinction). Specifically, participants were asked to rate each CXT−CS combination on a 5-point Likert scale: 1) Fear: “How scary is this?” (1 = not scary, 5 = very scary; Figs 1d, and 2) US expectancy: “Do you think that you will hear a loud sound with this?” (1 = definitely not, 5 = definitely yes; Fig. 1e). For physiological data, SCRs for each CS presentation were measured during each session and analyzed following our prior work^39^. Behavior was recorded for the CS period during the first half (‘early’) and second half (‘late) of each session (fear conditioning and extinction) in forward-to-backward distance from the CS (in virtual meters). Please see Supplemental Information for further detail.

### Statistical Analysis

Repeated-measures ANOVA was used to assess effects of CS-type (CS+, CS−) and time (first half/trial, second half/trial) on subjective ratings (fear, US expectancy), physiological responses (SCR), and behavioral action tendency (approach/avoidant) data within each session, following previous fear-extinction studies^7,39^. Pearson bivariate correlation was used to test for effects of age on conditioned fear measures, and for inter-correlations among conditioned fear measures (subjective, SCR, behavioral). All statistical analyses were performed in IBM SPSS Software v.24 using a *p* < 0.05 (two-tailed) statistical threshold. Effect sizes are reported for ANOVAs and *t*-tests, as η_p_^2^ and Cohen’s *d*, respectively.

## Electronic supplementary material

Supplementary Information
