# Supplementary material for: A novel paradigm to study interpersonal threat-related learning and extinction in children using virtual reality
Source: Sci Rep. 2017 Dec 4;7:16840. doi: 10.1038/s41598-017-17131-5 (PMC5715118; doi:10.1038/s41598-017-17131-5)
Supplement: Supplementary file 1 — Supplementary Information [file 41598_2017_17131_MOESM1_ESM.docx]

**Supplementary Information**

**A novel paradigm to study interpersonal threat-related learning and extinction in children using virtual reality**

Hilary A. Marusak, Craig A. Peters, Aneesh Hehr, Farrah Elrahal, and Christine A. Rabinak

**Supplemental Methods**

Advanced VR system

Participants were fitted with a high-resolution head mounted display (nVisor ST50, NVIS Inc.) that incorporates high-resolution color microdisplays with custom engineered optics to deliver unsurpassed visual acuity in a wide (50° diagonal) field-of-view format. The VR platform includes a Logitech joystick, a high-end rendering PC (Intel Xeon E5-1620 V2, Nvidia Geforce GTX 750 Ti, 8 GB RAM), the WorldViz VR Toolkit (Developer Edition), and an orientation tracker (OS3D, Inertial Labs). The VR system provides controlled and replicable experimental setups and allows for manipulations of environments. Virtual contexts and people were adapted from templates provided in the WorldViz VR Toolkit. Invisible boundaries restricted participant movement outside of the virtual hallway space, and visual boundaries restricted vision movement away from the CS. Earbud headphones (Sony) were used to standardize exposure to the US.

Cued fear-extinction paradigm with contextual modulations

Participants began by completing a brief practice tutorial (60 s) session in VR, wherein they received recorded instructions about how to navigate and explore the virtual environment. Then they were given the opportunity to move through space within a third virtual environment (indoor art gallery; not used in the experimental task) using a joystick in their dominant hand, as well as practice looking around the visual field by turning their head left and right (limited to 90 degrees with visual bounds while sitting in a stationary chair). They also practiced reporting the subjective ratings using the joystick, which they would need to make during the experimental task (described below; Figure 1c).

Following the practice tutorial, all participants underwent partial discrimination fear conditioning (~7.3 min). During fear conditioning, participants were presented with one CS on a computer screen (CS+; e.g., African American male) that co-terminated with the aversive white noise burst US through a pair of headphones at a partial reinforcement rate of 75% (i.e., partial reinforcement extinction effects^1,2^). A second CS (e.g., Asian male) was presented during fear conditioning but was never paired with the US (CS-). Fear conditioning consisted of 6 presentations of the CS+ that co-terminated with the US, intermixed with an additional 2 non-reinforced presentations of each the CS+, and 8 presentations of the CS-, all presented within the conditioning context (CXT+; Figure 1a).

After approximately a 10-min break all participants underwent an extinction session (~6.8 min) in which the CS+ was extinguished (presented in the absence of the US). There were 8 CS+ and 8 CS- trials presented in the extinction context (CXT-; Figure 1a).

At the beginning of each session, participants received recorded instructions to approach the person when they appeared, and that they may hear a loud sound: “During the game, you will meet virtual human beings. When they appear, you will be told to move directly towards them…During the game you might also hear a loud sound.” In both experimental sessions (fear conditioning and extinction) each trial began in a virtual hallway (CXT+ or CXT-) and participants were free to move through the virtual environment using the joystick. Of note, active navigation is thought to augment the spatial representation of contexts and more robustly engage hippocampal-dependent contextual learning^3^. After 3-8 s, the participant was set back to the start position (17 virtual meters from the CS) to control for differences in participant movement in the context. Concurrently, the CS rounded the corner, turned to face the participant, and a written prompt appeared at the lower part of the visual field that read “move toward the person”. The CS was facing the participant for 4 s. After 4 s, the trial terminated to a black screen (ITI; 4-9 s). The designation of the virtual avatars as CS+ or CS-, and the context as CXT+ or CXT- was counterbalanced across the participants. In addition, the order of trials was pseudo-randomized, such that no more than 2 presentations of the same avatar CS occurred in a row. Electrodes and headphones remained in place during all session (removed only during breaks).

Measures of Conditioned Fear

*Subjective - Fear and US expectancy ratings:* Participants reported subjective ratings at the beginning (‘start’), after the first half (‘early’), and after the second half (‘late) of each session (fear conditioning and extinction). Specifically, participants were asked to rate each CXT-CS combination on a 5-point Likert scale: 1) Fear: “How scary is this?” (1 = not scary, 5 = very scary; Figure 1d), and 2) US expectancy: “Do you think that you will hear a loud sound with this?” (1= definitely not, 5 = definitely yes; Figure 1e). As our focus was on cued fear-learning within a contextual environment, the questions were designed to be vague, to not lead the participant to focus on the CS in the absence of the CXT, or vice versa. Of note, ratings at the ‘start’ timepoint are available only for a subset of youth (n = 14).

*Physiological - Skin conductance response (SCR):* SCRs were measured using two electrodes (EL509, BIOPAC Systems, Inc., Goleta, CA.) attached between the first and second phalanges of the second and third digits of the non-dominant hand (left in all but 2 participants). AcqKnowledge software v.4.4 (BIOPAC Systems) was used to acquire the SCR trace (1000 samples per second) and to calculate SCRs using event-related electrodermal response analysis. The recorded waveforms were low pass filtered using a Blackman window (cutoff frequency = 25 Hz, coefficients = 160) and mean value smoothed over 100 adjacent data points prior to scoring. For unpaired CS trials (i.e., no US), SCRs were calculated on the maximum of the SCR trace within a standard 0.5-4.5 s latency window following CS onset, and accounting for a baseline (2 s window prior to CS onset)^4^. The minimal response criterion was 0.02 µS. Raw SCRs were square root transformed to normalize the distributions across participants. For paired CS-US trials during conditioning, we used a smaller window size (0-3.5 s latency) to assess the level of conditioned responding in anticipation of the aversive US separate from unconditioned responses to the noise bursts, themselves. Of note, this may have excluded peak SCRs to the CS+ that occurred after this window. Mean SCR to the US is also reported.

*Behavioral – Approach/avoidant behavioral action tendencies:* Participant movement following CS onset (3 s window, to avoid contamination from the US at 3.5 s) was recorded during the first half (‘early’) and second half (‘late) of each session (fear conditioning and extinction) in forward-to-backward distance from the CS (in virtual meters). Of note, although participants could move side-to-side in the virtual hallway, movement was relatively restricted within the physical confines of the hallway to balance the need for equivalent exposure to the CS across participants (i.e., participants could not limit their exposure to the CS by moving or turning away).

**References**

1. Milad, M. R. *et al.* Neurobiological Basis of Failure to Recall Extinction Memory in Posttraumatic Stress Disorder. *Biol. Psychiatry* **66,** 1075–1082 (2009).

2. Milad, M. R. *et al.* Recall of Fear Extinction in Humans Activates the Ventromedial Prefrontal Cortex and Hippocampus in Concert. *Biol. Psychiatry* **62,** 446–454 (2007).

3. Burgess, N., Maguire, E. A. & O’Keefe, J. The human hippocampus and spatial and episodic memory. *Neuron* **35,** 625–641 (2002).

4. Rabinak, C. A. *et al.* Cannabinoid modulation of prefrontal-limbic activation during fear extinction learning and recall in humans. *Neurobiol. Learn. Mem.* **113,** 125–134 (2014).
